# Expression of immune-response genes in lepidopteran host is suppressed by venom from an endoparasitoid, *Pteromalus puparum*

**DOI:** 10.1186/1471-2164-11-484

**Published:** 2010-09-02

**Authors:** Qi Fang, Lei Wang, Jiaying Zhu, Yanmin Li, Qisheng Song, David W Stanley, Zunnu-raen Akhtar, Gongyin Ye

**Affiliations:** 1 State Key Laboratory of Rice Biology & Key Laboratory of Molecular Biology of Crop Pathogens and Insects of Ministry of Agriculture, Institute of Insect Sciences, Zhejiang University, Hangzhou 310029, China; 2 Division of Plant Sciences, University of Missouri, Columbia, MO 65211, USA; 3 USDA/Agricultural Research Service, Biological Control of Insects Research Laboratory, Columbia, MO 65203, USA

## Abstract

**Background:**

The relationships between parasitoids and their insect hosts have attracted attention at two levels. First, the basic biology of host-parasitoid interactions is of fundamental interest. Second, parasitoids are widely used as biological control agents in sustainable agricultural programs. Females of the gregarious endoparasitoid *Pteromalus puparum *(Hymenoptera: Pteromalidae) inject venom along with eggs into their hosts. *P. puparum *does not inject polydnaviruses during oviposition. For this reason, *P. puparum *and its pupal host, the small white butterfly *Pieris rapae *(Lepidoptera: Pieridae), comprise an excellent model system for studying the influence of an endoparasitoid venom on the biology of the pupal host. *P. puparum *venom suppresses the immunity of its host, although the suppressive mechanisms are not fully understood. In this study, we tested our hypothesis that *P. puparum *venom influences host gene expression in the two main immunity-conferring tissues, hemocytes and fat body.

**Results:**

At 1 h post-venom injection, we recorded significant decreases in transcript levels of 217 EST clones (revealing 113 genes identified *in silico*, including 62 unknown contigs) derived from forward subtractive libraries of host hemocytes and in transcript levels of 288 EST clones (221 genes identified *in silico*, including 123 unknown contigs) from libraries of host fat body. These genes are related to insect immune response, cytoskeleton, cell cycle and apoptosis, metabolism, transport, stress response and transcriptional and translational regulation. We verified the reliability of the suppression subtractive hybridization (SSH) data with semi-quantitative RT-PCR analysis of a set of randomly selected genes. This analysis showed that most of the selected genes were down-regulated after venom injection.

**Conclusions:**

Our findings support our hypothesis that *P. puparum *venom influences gene expression in host hemocytes and fat body. Specifically, the venom treatments led to reductions in expression of a large number of genes. Many of the down-regulated genes act in immunity, although others act in non-immune areas of host biology. We conclude that the actions of venom on host gene expression influence immunity as well as other aspects of host biology in ways that benefit the development and emergence of the next generation of parasitoids.

## Background

In all developmental stages, insects are challenged by a broad range of natural enemies, including viruses, bacteria, fungi, protozoa as well as various metazoan parasites [1-3]. Insects have effective innate immune responses to contend with foreign invaders. Invasions by foreign organisms trigger several immune response signaling pathways, including Toll and IMD pathways [4]. These and other pathways lead to expression of immune-related genes [5]. Insect immune systems include physical barriers to invasion as well as cellular and humoral immune responses [4,6,7]. Cellular immunity involves direct interactions between hemocytes and invaders. These interactions begin immediately after an invasion is detected and they include phagocytosis, nodule formation and, in the case of large invaders such as parasitoid eggs, encapsulation [6]_. _Humoral responses include synthesis of antimicrobial peptides (AMPs) mainly by fat body cells and hemocytes. These peptides appear in the hemolymph of infected insects, 6-12 h post-infection and it has been suggested that these proteins serve a "mop-up" phase of responding to infections [8]. Humoral immunity also involves prophenoloxidase (pro-PO) activating cascades, which mediate blood coagulation and melanization [9]. The distinction between cellular and humoral immune reactions is a matter of convenience and somewhat artificial as there are substantial interactions between cellular and humoral immune responses [10].

Oviposition into a host haemocoel stimulates host immune responses. Non-permissive hosts effectively encapsulate and kill the parasitoid's eggs. However, hymenopteran parasitoids and their hosts have co-evolved sophisticated relationships. Parasitoids express virulence factors that act to impair or circumvent host immunity and thereby facilitate pre-imaginal development within the host [11-14]. These factors include polydnaviruses (PDVs), venoms, virus-like particles (VLPs), ovarian fluids and teratocytes [15,16]. Parasitoid/host interactions are not completely understood at the molecular level, although the roles of PDVs in impairing host defenses have received considerable attention [11,17]. As an example, the *Hyposoter didymator *ichnovirus (HdIV) influences several aspects of host immune functions, including gene regulation [18], impairing host encapsulation reaction [19], and reducing pro-PO activity [20,21]. The venom associated with injecting parasitoid eggs into their hosts is another virulence factor. Unlike the venom from spiders, scorpions or social hymenoptera, which cause neural paralysis and other pathological events [22], the venom from parasitoids disables host immunity and/or manipulates host physiology to create an environment favorable for the development of the parasitoid [15,20,23]. For instance, *Leptopilina boulardi *venom inhibits the host immune responses [12], due to immune suppressing factors in the venom, Rho-GAP protein [13] and serpin [24], which suppress host hemocyte changes and pro-PO cascade, respectively. *Nasonia vitripennis *venom contains a phenoloxidase (PO) activity that may be toxic to the host hemocytes [25]. Generally, there is less knowledge, particularly at the level of gene expression, on parasitoid venom relative to literature on PDVs.

The gregarious parasitoid, *Pteromalus puparum *is a pupal parasitoid of *Pieris rapae*, a vegetable pest worldwide. This parasitoid injects venom, but not PDVs, into its hosts during oviposition. *P. puparum *and its pupal host *P. rapae *comprise a model system for research into the influence of venom on host biology in non-PDV system [26,27]. *P. puparum *venom causes significant alteration in total number and morphology of host hemocytes, and inhibits host cellular immune responses [28]. Although these visible effects of the venom have been recorded, information on the molecular mechanisms of venom actions is lacking. To begin investigating this lack, we posed the hypothesis that *P. puparum *venom influences gene expression in the main immunity-conferring tissues of its host, the hemocytes and fat body. In this paper, we report on the outcomes of experiments designed to test our hypothesis.

## Results and Discussion

### Host encapsulation reaction inhibited by parasitoid venom

**Figure 1:**
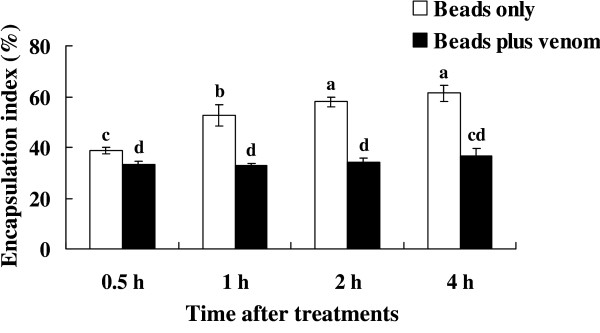
**Encapsulation analysis**. Encapsulation of Sephadex A-50 beads by *P. rapae *hemocytes. Each histogram represents the mean encapsulation index, and the error bar represents the standard deviation of each mean value (mean ± SD, *n *= 3). All raw data were transformed by arcsine square root before two-way ANOVA analysis. Histograms annotated with the same letter are not significantly different.

To statistically analyze the suppression effect of *P. puparum *venom on encapsulation by *P. rapae *hemcoytes, we created an index of encapsulation, using abiotic Sephadex A-50 beads as model parasitoid eggs [29]. The index of encapsulation is a proportion of Sephadex beads encapsulated to measured extents, from 1 to 5, described below in M&Ms. As shown in Figure 1, the index of encapsulation was significantly affected by treatments (*F *= 30.56; *df *= 3; *P *= 0.0001), sampling time post treatments (*F *= 338.33; *df *= 1; *P *= 0.0001) and treatment-sampling time interaction (*F *= 19.64; *df *= 3; *P *= 0.0001). Sephadex A-50 beads were encapsulated after they were injected into the host pupae, and the encapsulation increased from 0.5 hour to 4 hours after injection. The encapsulation indices after 2 and 4 h incubations were significantly higher than recorded after 0.5 and 1 h incubations. Following venom plus bead injection, the index of the host encapsulation was significantly decreased compared to bead-only injection. These results indicate that the influence of venom on encapsulation reactions occurred very quickly. Venoms from parasitic wasps, either alone or co-effective with other virulence factors, have diverse functions, especially in the interference of host immune response [11,15,20,23]. For some parasitoid species such as *Pimpla hypochondriaca *[30] and *Nasonia vitripenns *[31] (devoid of PDVs or other symbiotic viruses) venoms are likely the key active factor in immune suppression. Similarly, venom from *P. puparum *is an inhibitor of host immunity [29]. Based on these encapsulation-inhibiting results, we selected 1 h post injection (PI) for beads-only and beads plus venom for the "tester" and "driver" cDNA preparations, respectively.

### General overview of forward subtractive libraries prepared from hemocytes and fat body

**Figure 2:**
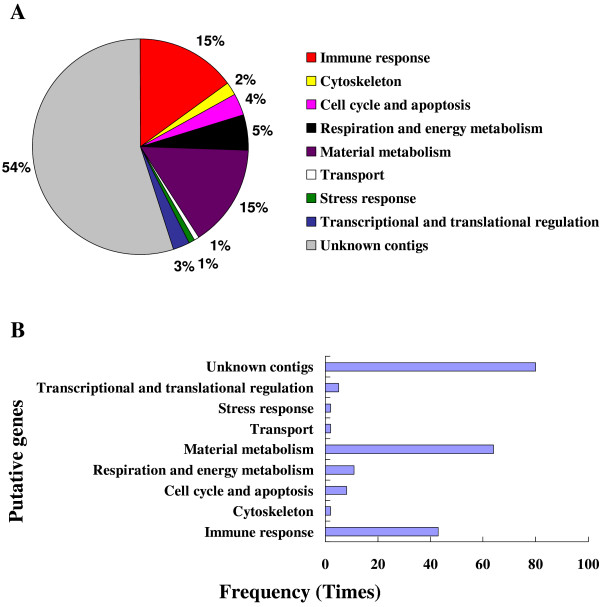
**Annotation of the genes identified in host hemocytes**. Annotation of the genes identified in host hemocyte forward SSH library. The identified genes were categorized into nine groups as indicated in the figure. (A): Gene transcriptional composition. (B): Gene frequency. The total number of the sequenced ESTs assigned into each group.

**Figure 3:**
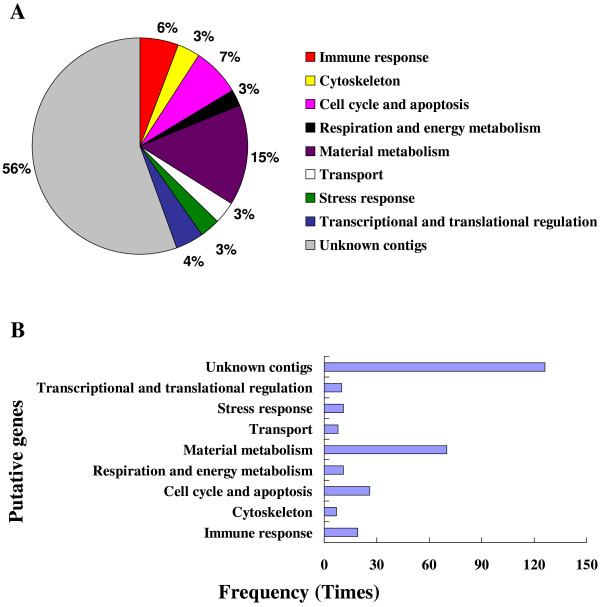
**Annotation of the genes identified in host fat body**. Annotation of the genes identified in host fat body forward SSH library. The identified genes were categorized into nine groups. (A): Gene transcriptional composition. (B): Gene frequency. The representation is described above.

**Figure 4:**
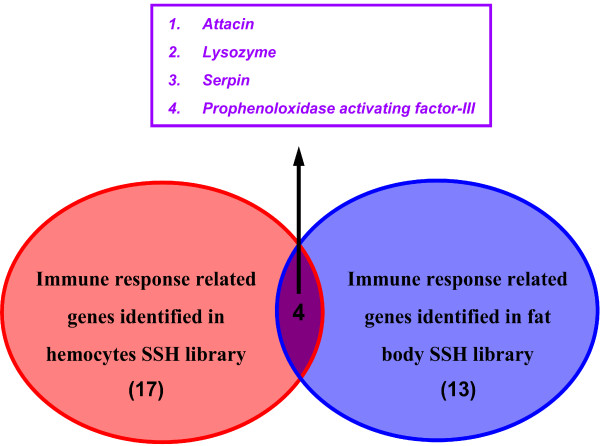
**Distribution of immune response related genes in hemocyte and fat body subtracted libraries**. Numbers in parentheses are total immune response related gene cluster from each library. Genes listed in the box occurred in both libraries. Details for the genes are listed in Tables 1 & 2.

A subtracted cDNA library was constructed using suppression subtractive hybridization (SSH). The transcript levels of these genes were higher in a pool of "tester" cDNAs (beads-only injection into the host) than those in a pool of "driver" cDNAs (beads plus venom injection). A total of 384 hemocyte clones and 480 fat body clones were randomly selected from forward SSH libraries and subjected to colony PCR and dot-blot hybridization (Additional File 1). For dot blot hybridization, mRNA samples from hemocytes and fat body (following beads-only and beads plus venom injections) were labeled by digoxigenin as two sets of probes in reverse transcriptional reactions. Hybridization was performed using two probes against the purified colony PCR product blots from two SSH libraries, respectively. If signals of the blots hybridized with the beads-only injection probe were stronger than signals with the venom plus beads injection probe, these clones were considered positive. A total of 225 hemocyte clones and 296 fat body clones were verified as positive clones, which were isolated and sequenced. Subsequently, 217 and 288 of available ESTs sequences were obtained from hemocytes and fat body libraries. The high quality and non-redundant sequences have been deposited in the EST database (dbEST) with GenBank accession numbers from GW316222 to GW316411 and GW316614 to GW316709. The obtained sequences were used to BLASTX interrogate the GenBank, EMBL and DDBJ databases via the National Center for Biotechnology Information. One hundred and thirteen and 221 genes identified *in silico *were down-regulated in *P. rapae *hemocytes and fat body, respectively, 1 h PI of parasitoid venom (Tables 1 &2). We assorted the genes into nine groups, including immune response, cytoskeleton, cell cycle and apoptosis, respiration and energy metabolism, material metabolism, transport, stress response, and transcriptional and translational regulation as well as unknown contigs (Figures. 2A & 3A). In hemocyte and fat body SSH libraries, 54% and 56% of the *in silico *identified genes, respectively, have either no significant similarity with database sequences or similarity with hypothetical proteins of unknown function. We used InterProScan to do an integrated search in PROSITE, Pfam, and PRINTS databases at the EMBL-European Bioinformatics Institute to seek functional domains of these unknown genes, finding no domains. The identified genes similar to known genes in database in high frequency belong to material metabolism and immune response groups in hemocyte library (Figure 2B). For the fat body library, the high frequency genes were assigned to material metabolism and cell cycle and apoptosis groups (Figure 3B). Genes and their frequency response after venom injection in hemocytes differed from those influenced in fat body (Tables 1 &2). In the venom treated hemocytes we found 17 immune-related transcripts (15% of the overall genes identified *in silico *from the hemocytes SSH library) that were negatively modulated (Figure 2A). For the venom treated fat body, 13 immune-related transcripts (only 6% from the overall *in silico *identified genes) were down regulated (Figure 3A). It appears that host hemocytes are the primary immunosuppressive targets for the venom. This is reasonable because encapsulation is the principle defense reaction to endoparasitoid invasions [6]. We identified *in silico *only four genes (attacin, lysozyme, pro-PO activating factor III and serpin; Figure 4) down-regulated by venom for both hemocytes and fat body. Whereas immune challenges promote gene up-regulation, particularly in humoral pathways, our SSH results show that many genes are down-regulated by venom. We recognize that down-regulation of a broad spectrum of genes may reflect a non-specific toxicity rather than mechanistic regulation of specific genes, a subject of future hypotheses. Nonetheless, SSH is not a genome-wide analysis, and it is possible that an even richer assembly of venom target genes were not detected.

**Table 1:** Identified genes from hemocyte subtracted library and their distribution in major functional categories

Classifications	EST ID	Matches	Organism	Predicted function^a^	E-Value
**Immune response**	hemo-115	AAD09288	*Hyphantria cunea*	Putative attacin	4e-13
	hemo-47	BAE78585	*Bombyx mori*	Immune inducible protein	4e-19
	hemo-101	BAG12297	*Samia cynthia*	Gallerimycin	1e-06
	hemo-36	AAT94287	*Artogeia rapae*	Antimicrobial peptide hinnavin II	6e-18
	hemo-35	AAT94286	*A. rapae*	Lysozyme II	2e-52
	hemo-363	AAW24481	*Spodoptera litura*	Prophenoloxidase activating enzyme 3	2e-12
	hemo-281	AAC64004	*Manduca sexta*	Prophenoloxidase activating enzyme precursor	1e-27
	hemo-327	AAW24480	*S. litura*	Prophenol oxidase activating enzyme 1	8e-17
	hemo-176	BAC15604	*Holotrichia diomphalia*	Prophenoloxidase activating factor-III	3e-10
	hemo-127	AAV91007	*Manduca sexta*	Hemolymph proteinase 9	2e-27
	hemo-20	AAD09285	*H. cunea*	Serpin	4e-39
	hemo-17	BAB79277	*Galleria mellonella*	Calreticulin	6e-27
	hemo-375	ABB92836	*Spodoptera frugiperda*	Scavenger receptor	3e-32
	hemo-46	CAB38429	*B. mori*	Lipopolysaccharide binding protein	5e-109
	hemo-357	BAA03124	*B. mori*	Lectin	6e-04
	hemo-53	EAT41228	*Aedes aegypti*	Clathrin coat assembly protein	2e-47
	hemo-172	XP_973182	*Tribolium castaneum*	N-acetylneuraminic acid phosphate synthase	4e-04
**Cytoskeleton**	hemo-232	ABF51446	*B. mori*	Actin-depolymerizing factor 1	6e-32
	hemo-158	XP_001606290	*Nasonia vitripennis*	Beta-1 tubulin	5e-43
**Cell cycle and Apoptosis**	hemo-11	BAA23126	*B. mori*	BmP109 related to apoptosis	8e-36
	hemo-15	ABK90824	*Spodoptera exigua*	Cathepsin L-like cysteine proteinase	1e-41
	hemo-19	P00037	*S. cynthia*	Cytochrome c	6e-55
	hemo-308	EDM11222	*Rattus norvegicus*	Phosphatidylserine-specific phospholipase A1	4e-16
**Respiration and Energy metabolism**	hemo-351	EAT45772	*A. aegypti*	Citrate synthase	1e-53
	hemo-141	CAA63422	*M. sexta*	Vacuolar ATP synthase subunit G	2e-12
	hemo-27	AAY89313	*Coreana raphaelis*	Cytochrome b	7e-76
	hemo-319	AAF98029	*Pieris napi*	Cytochrome c oxidase I (COI) gene	7e-70
	hemo-37	AAP42814	*Bombyx mandarina*	NADH dehydrogenase subunit 3	2e-43
	hemo-118	AAY89314	*C. raphaelis*	NADH-dehydrogenase subunit 1	4e-61
**Material metabolism**	hemo-167	ABC17802	*Oryctolagus cuniculus*	Pyrimidine 5'-nucleotidase	3e-10
	hemo-376	EAT38872	*A. aegypti*	Inosine-5-monophosphate dehydrogenase	1e-109
	hemo-32	EAT45824	*A. aegypti*	Skd/vacuolar sorting	8e-29
	hemo-5	AY351422	*Artogeia napi*	12 S ribosomal RNA gene	2e-95
	hemo-1	DQ351082	*P. napi*	16 S ribosomal RNA gene	0.0
	hemo-170	AAL26579	*S. frugiperda*	Ribosomal protein S3A	2e-90
	hemo-263	AAV34860	*B. mori*	Ribosomal protein S4	2e-63
	hemo-257	AAA20402	*M. sexta*	Ribosomal protein S7	2e-94
	hemo-314	AAK92188	*S. frugiperda*	Ribosomal protein S19	5e-67
	hemo-117	ABS57440	*Heliconius melpomene*	Ribosomal protein L7a	2e-88
	hemo-273	AAK76990	*S. frugiperda*	Ribosomal protein L10A	9e-22
	hemo-239	AAV34822	*B. mori*	Ribosomal protein L11	2e-28
	hemo-288	BAD18973	*Antheraea yamamai*	Ribosomal protein L13	7e-51
	hemo-155	AAV34827	*B. mori*	Ribosomal protein L15	7e-45
	hemo-161	BAD26655	*Plutella xylostella*	Ribosomal protein L27A2	9e-35
	hemo-371	AAL26577	*S. frugiperda*	Ribosomal protein L29	1e-23
	hemo-126	BAD93614	*B. mori*	Protein disulfide-isomerase like protein	9e-61
**Transport**	hemo-109	XP_975236	*T. castaneum*	Transmembrane protein 41B	2e-69
**Stress response**	hemo-247	ABF51296	*B. mori*	Peroxiredoxin	3e-08
**Transcriptional and Translational regulation**	hemo-337	ABF51438	*B. mori*	Transcription initiation factor IIA	6e-16
	hemo-128	XP_972330	*T. castaneum*	Eukaryotic translation initiation factor 2A	4e-31
	hemo-102	ABF51379	*B. mori*	Eukaryotic translation initiation factor 4A	5e-42
**Unknown contigs**	62 ESTs				

*^a ^*Predicted function: annotation of the subtracted cDNA sequences was based on GenBank/EMBL/DDBJ database using the BLASTX program.

**Table 2:** Identified genes from fat body subtracted library and their distribution in major functional categories

Classifications	EST ID	Matches	Organism	**Predicted function**^**a**^	E-Value
**Immune response**	FB-212	AAX51192	*Helicoverpa armigera*	Attacin	7e-40
	FB-9	BAA22884	*Bombyx mori*	Lebocin 4	5e-17
	FB-65	AAT94286	*Artogeia rapae*	Lysozyme II	3e-28
	FB-226	P85214	*Galleria mellonella*	Proline-rich antimicrobial peptide 1	3e-04
	FB-254	NP_001040277	*B. mori*	Salivary cysteine-rich peptide	5e-30
	FB-42	AAV91013	*Manduca sexta*	Hemolymph proteinase 16	2e-28
	FB-71	AAV91003	*M. sexta*	Hemolymph proteinase 5	7e-42
	FB-264	BAC15604	*Holotrichia diomphalia*	Prophenoloxidase activating factor-III	3e-09
	FB-224	AAD09285	*Hyphantria cunea*	Putative serpin	4e-38
	FB-286	AAD09290	*H. cunea*	Gram negative binding protein	2e-43
	FB-178	ABF51394	*B. mori*	S-adenosyl-L-homocysteine hydrolase	1e-82
	FB-23	ABG72723	*Antheraea mylitta*	Protease inhibitor-like protein	3e-13
	FB-215	XP_393519	*Apis mellifera*	Fatty acid-binding protein	3e-15
**Cytoskeleton**	FB-37	ABF51271	*B. mori*	Transgelin	9e-39
	FB-44	ABF51380	*B. mori*	F-actin capping protein beta subunit	1e-36
	FB-48	ABF51490	*B. mori*	Troponin C	2e-72
	FB-94	ABF51254	*B. mori*	Microtubule-associated protein	4e-31
	FB-136	DQ443357	*B. mori*	Actin-depolymerizing factor 1 mRNA	3e-28
	FB-152	AAT99314	*B. mori*	Profilin	2e-23
	FB-183	XP_001604074	*Nasonia vitripennis*	Rho GDP-dissociation inhibitor	2e-34
**Cell cycle and Apoptosis**	FB-41	EDS32890	*Culex pipiens quinquefasciatus*	Testis/seletal muscle specificty phosphatase	1e-21
	FB-150	EDS41027	*C. pipiens quinquefasciatus*	Laminin subunit alpha	2e-30
	FB-50	XP_971047	*Tribolium castaneum*	Der1-like domain family, member 2	4e-77
	FB-56	BAD52259	*Plutella xylostella*	Receptor for activated protein kinase C	1e-27
	FB-130	BAC10621	*B. mori*	Pleiotrophin-like protein	7e-30
	FB-205	ABF18450	*Aedes aegypti*	Growth and transformation-dependent protein	7e-19
	FB-257	BAG30771	*Papilio xuthus*	SPARC	8e-53
	FB-36	BAA23126	*B. mori*	BmP109	4e-106
	FB-200	NP_001036881	*B. mori*	Annexin B13	2e-65
	FB-79	ABE72972	*A. aegypti*	Cathepsin L	1e-37
	FB-102	ABF51517	*B. mori*	Legumaturain	5e-49
	FB-75	NP_001106229	*B. mori*	Mod(mdg4)-heS00531	1e-53
	FB-7	ABD36346	*B. mori*	GTP-binding nuclear protein Ran	1e-64
	FB-61	EAT45318	*A. aegypti*	Gtpase-activating protein	2e-95
	FB-45	L03281	*M. sexta*	Met-rich storage protein SP1A	1e-41
	FB-276	Q06342	*Trichoplusia ni*	Basic juvenile hormone-suppressible protein	4e-31
**Respiration and Energy metabolism**	FB-30	ABB90008	*Colias eurytheme*	Glyceraldehyde-3-phosphate dehydrogenase	2e-139
	FB-43	P31402	*M. sexta*	Vacuolar proton pump subunit E	2e-48
	FB-210	NP_001040257	*B. mori*	Cytosolic malate dehydrogenase	1e-91
	FB-230	AAW39031	*Danaus ismare*	Cytochrome c oxidase subunit I	1e-64
	FB-124	ACB49306	*Artogeia melete*	Cytochrome c oxidase subunit III	2e-88
	FB-211	ABD36116	*B. mori*	Electron-transfer-flavoprotein beta polypeptide	3e-93
**Material metabolism**	FB-76	ABA00463	*B. mori*	Phosphoglyceromutase	7e-60
	FB-70	XP_001655220	*A. aegypti*	N-acetylglucosamine-6-phosphate deacetylase	1e-13
	FB-231	NP_001040128	*B. mori*	Glucosamine-6-phosphate N-acetyltransferase	9e-89
	FB-40	XP_973042	*T. castaneum*	L-3-hydroxyacyl-CoA dehydrogenase	2e-48
	FB-157	AAT72922	*Spodoptera littoralis*	Sterol carrier protein	3e-23
	FB-20	AAG34698	*Epiphyas postvittana*	Apolipophorin-III-like protein	5e-28
	FB-33	AAT76806	*G. mellonella*	Apolipophorin	3e-24
	FB-260	DQ482581	*G. mellonella*	Lipophorin receptor mRNA	6e-38
	FB-168	AAG44960	*Corcyra cephalonica*	Hexamerin 2	3e-70
	FB-66	BAA34090	*B. mori*	Arylphorin	8e-24
	FB-78	CAB55603	*Spodoptera litura*	Moderately methionine rich storage protein	9e-103
	FB-22	DQ351082	*Pieris napi*	16 S ribosomal RNA gene	0
	FB-15	AAV34860	*B. mori*	Ribosomal protein S4	1e-31
	FB-52	ABG81962	*Diaphorina citri*	Putative S5e ribosomal protein	6e-49
	FB-144	Q94624	*M. sexta*	40 S ribosomal protein S6	4e-105
	FB-57	AAV34865	*B. mori*	Ribosomal protein S9	1e-67
	FB-199	AAK92188	*Spodoptera frugiperda*	Ribosomal protein S19	4e-41
	FB-104	CAD25759	*Encephalitozoon cuniculi*	40 S ribosomal protein S23	2e-37
	FB-148	AAL62468	*S. frugiperda*	Ribosomal protein L3	4e-29
	FB-134	ABS57444	*Heliconius melpomene*	Ribosomal protein L12	2e-56
	FB-101	AAV34826	*B. mori*	Ribosomal protein L14	3e-83
	FB-34	AAG45936	*B. mori*	Protein disulfide isomerase	2e-86
	FB-100	BAD93614	*B. mori*	Protein disulfide-isomerase like protein	5e-79
	FB-49	EAT43532	*A. aegypti*	Seryl-tRNA synthetase	1e-88
	FB-95	ABD36309	*B. mori*	Homocysteine S-methyltransferase	6e-31
	FB-53	XP_001866517	*C. pipiens quinquefasciatus*	26 S protease regulatory subunit 6A	6e-89
	FB-204	EAT35416	*A. aegypti*	26 S proteasome subunit S9	3e-21
	FB-86	XP_391945	*A. mellifera*	Proteasome p44.5 subunit	8e-30
	FB-120	XP_001897032	*Brugia malayi*	Polyubiquitin precursor	5e-35
	FB-234	EAT39130	*A. aegypti*	Ubiquitin fusion degradaton protein	1e-17
	FB-247	EDP34630	*B. malayi*	Ubiquitin	2e-24
	FB-273	ABF51361	*B. mori*	Zinc-containing alcohol dehydrogenase	6e-59
	FB-3	AAF23078	*Choristoneura fumiferana*	Glutathione S-transferase	2e-73
**Transport**	FB-91	BAD27263	*Chilo suppressalis*	Transferrin	2e-110
	FB-256	P22297	*M. sexta*	Transferrin precursor	5e-116
	FB-208	ABM92425	*Pieris rapae*	Ferritin HCH	7e-89
	FB-2	XP_001605887	*N. vitripennis*	Mitochondrial carrier protein ymc	9e-35
	FB-14	XP_968248	*T. castaneum*	Outer mitochondrial membrane translocase	2e-30
	FB-265	AAS91007	*B. mori*	Kiser	1e-93
	FB-228	ABF51232	*B. mori*	Transmembrane trafficking protein	7e-39
**Stress response**	FB-113	CAD26198	*E. cuniculi*	Heat shock related 70kDa protein	1e-38
	FB-115	ABM88156	*Plodia interpunctella*	Heat shock cognate 70	5e-101
	FB-263	BAE48744	*P. xylostella*	Heat shock protein 19.5	3e-25
	FB-128	ABR27869	*Triatoma infestans*	DNA-binding nuclear protein p8	4e-19
	FB-60	AAY82465	*B. mori*	Ribosome-associated membrane protein 4	3e-28
	FB-138	ABD36103	*B. mori*	DnaJ-like protein	3e-16
	FB-269	XP_969600	*T. castaneum*	Peroxidase precursor	8e-35
**Transcriptional and Translational regulation**	FB-35	XP_623868	*A. mellifera*	ATP-dependent helicase TfIIF-beta	8e-43
	FB-255	AAT51707	*C. fumiferana*	DEAD box RNA helicase	3e-19
	FB-25	ABF51379	*B. mori*	Eukaryotic translation initiation factor 4A	2e-49
	FB-114	NP_001086988	*Xenopus laevis*	Eukaryotic translation initiation factor 2C	3e-32
	FB-252	ABF71565	*B. mori*	Translation elongation factor 2	1e-117
	FB-99	BAB16696	*B. mori*	En10 protein	1e-91
	FB-68	AAZ31205	*Delias nais*	Elongation factor-1 alpha	2e-55
	FB-81	BAB21109	*B. mori*	Elongation factor 1 delta	9e-26
	FB-218	ABH10804	*B. mori*	Signal recognition particle 68 kDa protein	6e-31
**Unknown contigs**	123 ESTs				

*^a ^*The representation is same as the footnote of the Table 1.

### Genes related to immune defense

#### Genes encoding proteins involved in the insect humoral immune response

##### Antimicrobial peptides

Ten potential antimicrobial molecules have lower transcript levels in hemocytes or fat body of the venom injected hosts, including cecropin, lysozyme, attacin, lebocin, proline-rich AMP, cysteine-rich peptide, gallerimycin and immune inducible peptide (Tables 1 &2). For screening the two SSH libraries, the full cDNA length sequences of cecropin and lysozyme of *P. rapae *were obtained (Additional files 2 & 3), and we found three isoforms of the cecropins and one of lysozyme in two subtractive cDNA libraries. Cecropins have also been identified from dipterans [32], tunicates [33] and nematodes [34], which have residues in the consensus region of lepidopteran cecropins. Our multiple sequence alignment and phylogenetic analysis (Additional file 2) shows that the *P. rapae *cecropins are virtually unrelated to worm cecropins, more closely related to other lepidopteran cecropins and distant from dipteran cecropin. Lysozyme is a ubiquitous enzyme involved in self-defense from bacterial infection [35]. Our results indicate that the lysozyme mRNA is present in *P. rapae *hemocytes and fat body. Again, our multiple sequence alignment and phylogenetic analysis (Additional file 3) shows that the *P. rapae *lysozme is related to other lepidopteran lysozymes. The activity of host lysozyme is down-regulated by parasitization [21]. For example, parasitization by *Campoletis sonorensis*, down-regulated plasma lysozyme activity in *Heliothis virescens *[36]. Ours is the first report of a transcriptional down-regulation of these antimicrobial genes due to endoparasitoid venom. Down-regulation of the immune response genes expression in the host may be a protective mechanism for parasitoid eggs.

##### The prophenoloxidase cascade

Our results indicate that venom down-regulated the pro-PO cascade system by directly interfering with transcript levels of genes encoding proteins involved in activating pro-PO. We found four *P. rapae *sequences named Pr-PPAEs, similar to the pro-PO activating enzymes/factors (PPAEs/PPAFs) in other lepidopterans, three sequences named Pr-HPs similar to hemolymph proteinases (HPs) of *Manduca sexta*, as well as one named Pr-serpin similar to serine proteinase inhibitor (serpin) of *Hyphantria cunea*, are down-regulated in the hemocytes or fat body 1 h PI (Tables 1 &2). Insect PO is produced from an inactive zymogen (pro-PO) by proteolytic cleavage. This process is mediated by a proteinase cascade plus additional factors, including immulectins [17]. The PPAEs are serine proteinases (SPs) which directly cleave or indirectly activate the pro-PO precursor. In *M. sexta*, transcription of the pro-PO activating proteinase-1 (PAP-1) gene is up-regulated in both hemocytes and fat body in response to bacterial induction and down-regulated by treatment with 20-hydroxyecdysone. This PAP-1 gene may be under control of immune and hormonal signals [37]. Pr-HPs identified in our study belong to the SP superfamily and would likely take part in the pro-PO activating system. Recently, a set of HPs have been identified and characterized in *M. sexta*, of which HP9 and HP6 could be induced by immune-challenge both in hemocytes and fat body [38]. In our study, the transcript levels of Pr-PPAEs and Pr-HPs were down-regulated by venom. We identified one serpin from *P. rapae *hemocytes and fat body, which may be a regulator of SPs, including some PPAEs [39]. When some PPAEs are activated by immune-challenge, the serpins co-express to reduce the expression and activity of PPAEs. This protects the host, because high PO activity is harmful to it. Taken with results of enzyme assays (last section, below), we suggest that venom may curtail melanization by down-regulating the transcript levels of genes encoding PPAEs, HPs, and serpin, but not the pro-PO gene directly.

#### Genes encoding proteins involved in the insect cellular immune response and/or non-self recognition

##### Lectins and gram negative binding protein

Venom treatment down-regulated a set of host genes with a high similarity to the lepidopteran lectins and the gram negative binding protein (GNBP), including *Bombyx mori *lipopolysaccharide binding protein [40], immulectin and the GNBP of *H. cunea *[41] (Tables 1 &2). Lepidopteran lectins are typical C-type lectins, with carbohydrate recognition domains. They function as pathogen recognition receptors, and the promoters of pro-PO activation in hemolymph [42], hemocyte nodule formation [40] and encapsulation [43]. As described for *Spodoptera frugiperda *[17], this *P. rapae *C-type lectin gene was identified in the host hemocyte SSH library, unlike other immulectins which are synthesized in the fat body and secreted into the hemolymph [44]. Down-regulation of the *P. rapae *lectins and GNBP homologue by parasitoid venom may be one mechanism of inhibiting egg encapsulation, observed in parasitized host pupae.

##### Calreticulin

In hemocytes, the transcript level of the calreticulin (CRT) gene was down-regulated by venom injection (Table 1). CRT is a conserved multifunctional Ca^2+ ^binding protein, present in a variety of cellular compartments [45]. Intra-cellular CRT functions as a molecular chaperone and regulator of Ca^2+ ^homeostasis. CRT is also found on the surface of cells, where it might participate in the processes of cellular adhesion and migration [46], inter-cellular signal transduction [45], or elimination of apoptotic cells [47]. CRT of *Galleria mellonella *was isolated from the soluble fraction of hemocyte lysates and could surround DEAE beads. CRT in hemocyte membranes participates in the non-self recognition in early-stage of the encapsulation. CRT may enhance the early-stage of the encapsulation [48]. CRT is on the surface of *P. rapae *hemocytes during the phagocytosis of yeast cells [49]. The transcript level of the host CRT gene is reduced by *P. puparum *venom and we speculate it suppresses encapsulation or non-self recognition.

##### Scavenger receptor

**Figure 5:**
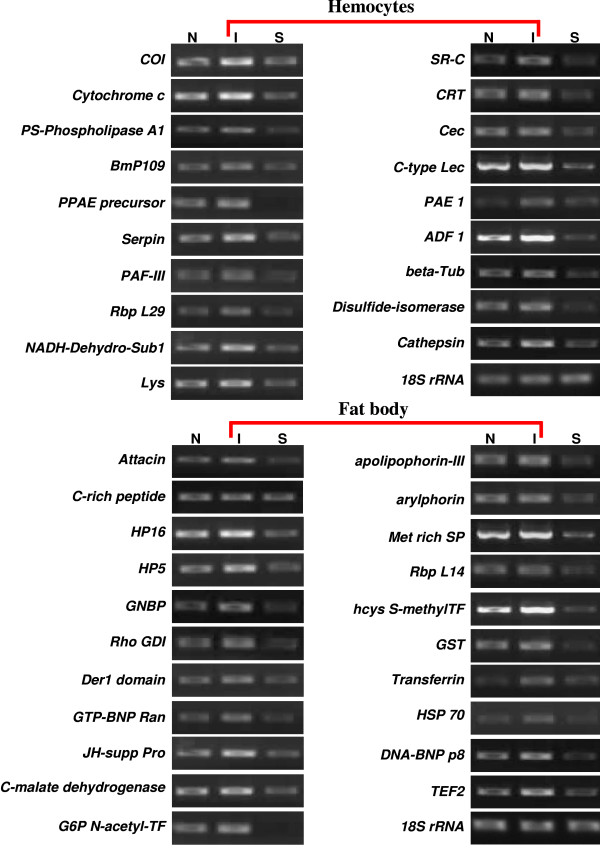
**Expression profiles of 40 candidate genes**. Expression profiles of 40 Immune-related candidate genes (19 from hemocytes SSH library and 21 from fat body SSH library) in host immune tissues. These genes were analyzed by semi-quantitative RT-PCR, using the *P. rapae 18 S rRNA *as a reference gene. The EST ID of the genes and the PCR primer sequences are given in additional file 4. Gene expression in host hemocytes and fat body cells at 1 h PI, "N" represents the negative control, treatment with Pringle's saline buffer. "I" represents the immune-inducible genes following Sephadex bead injection, and "S" denotes the immune-suppressed following venom treatment. Hemocytes and fat body genes are identified by the red lines.

One host gene down-regulated in response to the venom injection encodes a protein possessing a significant similarity with scavenger receptor (SR)-C like protein of *S. frugiperda *(Table 1). The *P. rapae *SR-C is predicted to be a trans-membrane protein, which possess a MAM domain and two tandem complement control protein (CCP) domains. In *Drosophila*, based on the functional characterization of SRs, MAM and CCP domains are sufficient for bacterial binding in phagocytosis [50], from which it appears that lepidopteran SRs act in phagocytosis [17]. Phagocytosis of *Spodoptera littoralis *granular hemocytes was suppressed by polyinosinic acid, a specific ligand of SRs [51]. SR-C may attach bacterial cells to lepidopteran hemocytes [17]. We found the transcript level of this gene was up-regulated by bead challenge at 1 h PI of Sephadex-A 50 beads (Figure 5). We suggest that SR-C influences phagocytosis and hemocyte encapsulation of parasitoid eggs.

##### Other molecules

Some of the down-regulated genes are potentially involved in other physiological processes. For example, venom treatment can alter the transcript levels of the annexin B gene in *P. rapae *(Table 2). Annexin B is a calcium binding protein that acts in functions such as membrane fusion, cell proliferation and differentiation, as well as cell apoptosis [52]. Particularly in *Drosophila*, annexin IX is an immune-inducible gene up-regulated in response to bacterial challenge [53]. The parasitoid venom could lead to disruption of the cell cytoskeleton in the host [11]. Besides down-regulation of the beta-tubulin subunit in the host immune system, our results show that venom injection influences transcript levels of several genes related to the actin-based motility actions. These include actin-depolymerizing factor 1, transgelin, troponin, microtubule-associated protein, F-actin capping protein, and profilin (Tables 1 &2). It may be inferred from these results that venom inhibition of the transcript levels of these genes may be related to cell migration, differentiation, or proliferation.

### *In silico *identified genes involved in transcription and protein biosynthesis

The expression of genes related to transcription and translation mechanisms are inhibited by venom. These include transcription initiation factor, eukaryotic translation initiation factors, RNA helicase and translation elongation factor (Tables 1 &2). The expression of genes involved in transcription and translation mechanisms are usually up-regulated by immune challenge in insect hemocytes or human monocytes [54]. The venom-related down-regulation of transcription and translation may be related to the suppression of host immune responses. Expression of genes similar to large and small subunits of ribosomal proteins, disulfide-isomerase-like protein and several proteins in the ubiquitin-proteasome pathway (Tables 1 &2) were inhibited by the venom treatment. These genes are related to synthesis, modification, or degradation of proteins. This may be related to host immunity because members of the ubiquitin- proteasome pathway act in cellular processes including regulation of cell cycle, division, development, differentiation, apoptosis, cell trafficking, and modulation of immune responses [55].

### Genes related to detoxification and stress response

Venom injection led to down-regulation of several transcripts encoding proteins potentially involved in detoxification and stress responses. These include genes similar to peroxiredoxin of *B. mori*, heat-shock protein 19.5 of *Plutella xylostella*, DnaJ-like protein of *B. mori*, and glutathione S-transferase (GST) of *Choristoneura. fumiferana *(Tables 1 &2). Peroxiredoxin is from hemocytes and fat body and the others come from fat body. We speculate that fat body is more active in detoxification and stress responses than hemocytes. Growth and development of endoparasites certainly stress their hosts, and it may be expected that *P. puparum *venom inhibits expression of these stress response genes.

### Semi-quantitative RT-PCR and enzyme assays results

We confirmed the transcript levels of the screened genes by semi-quantitative RT-PCR (Figure 5). The RNA templates were isolated from hemocytes and fat body of negative control, immune-induced, and immune-suppressed pupae, relative to a fragment of 18 S rRNA gene (Additional file 4). Transcript levels of most selected genes were induced by the Sephadex A-50 injection but not by phosphate buffer. We noted high expression of a few of the genes in negative controls. This is probably because wounding during injection induces expression of some genes. Most of the transcript levels of the selected immune-response genes were down-regulated following injection of the venom plus beads, except for the C-rich peptide transcript. Host genes encoding hemocyte PPAE precursor and SR-C were strongly down-regulated following venom injection.

**Figure 6:**
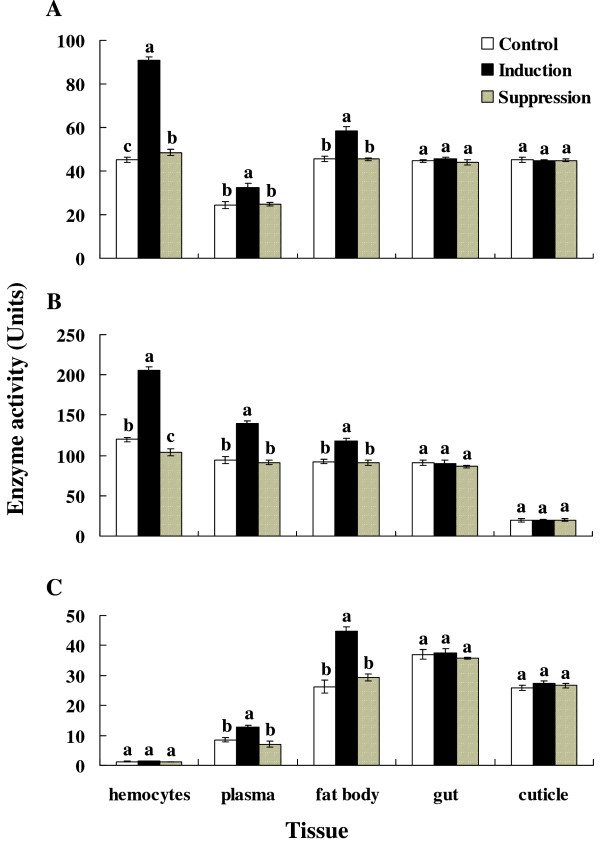
**Differential activities of enzymes**. Activities of antimicrobial and detoxification enzymes following challenge with buffer (negative control, white bars), Sephadex beads (immune challenge, black bars) and Sephadex beads plus venom (immune suppression, patterned bars). The indicated tissues were prepared for enzyme assays at 1 h PI. Panel (A) shows PO activity, panel (B) shows lysozyme activity and panel (C) shows GST activity. The units of enzyme activities are defined in M&Ms. Values are means ± SD. Histogram bars annotated with the same letter are not significantly different for each tested tissue.

We tested whether reduced mRNA levels of PPAEs, Lysozyme and GST resulted in similarly reduced protein levels by measuring activity of these enzymes (Figure 6). Bead challenges led to significant increases in lysozyme and PO activities in hemocytes (For PO, *F *= 957.71; *df *= 2, 6; *P *= 0.0001; for lysozyme, *F *= 571.15; *df *= 2, 6; *P *= 0.0001), plasma (For PO, *F *= 19.41; *df *= 2, 6; *P *= 0.0024; for lysozyme, *F *= 167.75; *df *= 2, 6; *P *= 0.0001) and fat body (For PO, *F *= 76.86; *df *= 2, 6; *P *= 0.0001; for lysozyme, *F *= 82.48; *df *= 2, 6; *P *= 0.0001), but not in gut (For PO, *F *= 2.51; *df *= 2, 6; *P *= 0.1618; for lysozyme, *F *= 1.76; *df *= 2, 6; *P *= 0.2497) and cuticle (For PO, *F *= 0.10; *df *= 2, 6; *P *= 0.9080; for lysozyme, *F *= 0.11; *df *= 2, 6; *P *= 0.8968). Bead challenge also led to significant increases in GST activity in plasma (*F *= 39.87; *df *= 2, 6; *P *= 0.0003) and fat body (*F *= 115.02; *df *= 2, 6; *P *= 0.0001), but not the other tissues (For hemocytes, *F *= 1.35; *df *= 2, 6; *P *= 0.3272; for gut, *F *= 1.79; *df *= 2, 6; *P *= 0.2452; for cuticle, *F *= 2.03; *df *= 2, 6; *P *= 0.2121). All three enzyme activities were not increased significantly following injection of beads plus parasitoid venom, comparing to negative controls. The results of SSH and RT-PCR experiments are parallel. We speculate that the parasitoid venom influences the upstream transcriptional down-regulation of gene expression.

## Conclusions

The data reported in this paper support our hypothesis that *P. puparum *venom influences gene expression in its pupal host, *P. rapae*. The following points apply. First, at the level of observable immune reactions, venom treatment rapidly leads to significant reductions in hemocytic encapsulation reactions to beads. Second, venom treatment led to down-regulation of induced gene transcript levels in the hemocytes and fat body of host pupae. Third, the observed gene down-regulation was confirmed by RT-PCR and enzyme assays. Taken together, these points bolster our hypothesis and they provide some insight into the molecular mechanism of the *P. puparum*/*P. rapae *parasitoid system. While there is very little literature on the chemical makeup of parasitoid venoms, we infer from the wide range of effects *P. puparum *venom exerts on its host that the venom is a complex substance with a large, albeit unknown, number of components. One direction of our research is to investigate the number and nature venom components.

## Methods

### Insect rearing

*P. rapae *were collected primarily from cabbage fields in the experimental farmland of Zhejiang University, Hangzhou, China. The laboratory host colony and the *P. puparum *colony were maintained as described previously and used in all experiments [56]. Briefly, the host larvae were fed on fresh cabbage leaves within a stainless steel-mesh cage (55 cm × 55 cm × 55 cm, 1.0 mm × 1.0 mm mesh at 25 ± 1°C, L: D = 10: 14 h) until they pupated. Freshly pupated hosts were exposed to mated female wasps in a glass tube (50 × 230 mm) for 48 h. The parasitized pupae were individually held in glass vials (18 × 82 mm) under the same conditions just described for the hosts. After emerging, the female wasps were collected and held in glass containers (also under the conditions just described), fed *ad lib *on 20% (v/v) honey solution to lengthen life span for 3-4 days until dissection of the venom reservoir and gland.

### Crude venom preparation

Adult wasps aged 3-4 days were used for venom collection as described by Wu et al [29]. Briefly, the venom reservoir and gland were dissected from the female reproductive system on an ice-cold convex slide, containing a drop of phosphate buffer [10 mM sodium phosphate (pH 8.0), 0.9% (w/v) NaCl, 15% (w/v) sucrose, 1 mM ethylene diamine teraacetic acid, and 1 mM phenylmethylsulfonyl fluoride] [57]. Five hundred glands and reservoirs were then transferred to a sterilized 1.5 milliliter Eppendorf tube and centrifuged at 12,000 g for 20 min at 4°C. The supernatant was collected as the crude venom and then filtered through a 0.22 micrometer cellulose acetate filter. The filtered crude venom was stored at -70°C until use. The crude venom solution was diluted with Pringle's phosphate-buffered saline (PBS) to the final concentration of 2 venom reservoir equivalents (VREs)/μl immediately before use.

### *In vivo *encapsulation assay

To assay the *in vivo *encapsulation in *P. rapae *pupa, Sephadex A-50 beads (GE healthcare, Piscataway, NJ) were used as *P. puparum *mimic eggs. We sterilized 0.5 mg beads by UV radiation in 1.0 ml PBS and transferred the solution into a sterilized 1.5 ml Eppendorf tube to prepare a stock solution. The stock solution was diluted to a concentration of approximately 100 beads/μl PBS containing 50 units/ml penicillin/streptomycin (Invitrogen, Carlsbad, CA) before injection. A half microliter of the diluted bead suspensions (approximately 50 beads in 0.5 μl PBS) was injected into newly pupated and immunologically naive host pupae, using a sterilized 801 RN micro-syringe (Hamilton Bonaduz AG, Bonaduz, Switzerland). The beads were recovered from the treated pupae at 0.5, 1, 2 and 4 h PI and analyzed for encapsulation. The beads were recovered by puncturing the pupal cuticle at the elytrum with a sterilized dissecting pin, and then collecting the hemolymph into a sterilized 1.5 ml Eppendorf tube charged with a few crystals of 1-phenyl-2-thiourea (PTU) (Sigma, Taufkirchen, Germany) by micropipette. 0.1 ml aliquots of the collected hemolymph with the encapsulated beads were immediately transferred into wells of polyvinyl 24-well microplates (NUNC, Roskilde, Denmark), which contained 10 μl of PTU (25%) and 10 μl anticoagulant solution (0.9% NaCl, 0.942% KCl, 0.082% CaCl_2_, 2% EDTA), then analyzed for encapsulation.

For experiments on suppression of encapsulation by parasitoid venom, 0.5 μl of crude venom solution (equal to 1 VRE) was co-injected with 0.5 μl of the diluted bead suspensions into a host recipient. After the same incubation periods PI, the beads were recovered and analyzed for encapsulation.

The total number of encapsulated beads and their encapsulation grades were observed under a phase contrast microscope (Leica, Wetzlar, Germany) at 400X, recorded and expressed as an encapsulation index [29]. Briefly, encapsulated beads were classified as 5 grades and the encapsulation index (%) = {∑ (the number of beads with a defined encapsulated grade × its corresponding grade number)/total number of beads observed × 5)} × 100. The observations were repeated three times, each one used separate venom preparations and 5 host pupae.

### Isolation of RNA from immune challenged and vemon down-regulated *P. rapae *pupae

One group of pupae (n = 50) were challenged with approximately 50 Sephadex A-50 beads to stimulate immune gene expression, and another group (n = 50) were challenged with co-injection of approximately 50 Sephadex A-50 beads plus venom (1 VRE), as described. At 1 h PI, hemolymph was collected from bead-challenged pupae into the same 2.0 ml Eppendorf tube (sterilized, RNase-free and containing a few crystals of PTU). Hemolymph was similarly collected from bead plus venom-challenged pupae into another tube. To prepare hemocytes, the hemolymph was centrifuged at 200 g for 10 min at 8°C, a procedure proven safe for hemocyte collection [29]. In a parallel procedure, fat body was directly isolated from each group of pupae (n = 10). The collected fat body and hemocyte tissues were then homogenized in liquid N_2 _and total RNA was extracted using the TRizol reagent (Invitrogen) according to the manufacturer's instruction. RNA integrity was confirmed by ethidium bromide gel staining and RNA quantity was determined spectrophotometrically at A _260/280_.

### Subtracted cDNA libraries construction and sequencing

Two micrograms of total RNA from the immune-challenged and venom-treated hemocytes and fat body were used as the original template, respectively. The SMART PCR cDNA Synthesis Kit (Clontech, Mountain View, CA, USA) was used to synthesize double-stranded cDNA, according to the manufacturer's instruction. Two kinds of SSH was performed for the synthesized cDNAs, using a PCR-Select cDNA Subtraction Kit (Clontech), according to the manufacturer's instruction.

In brief, we designated the fat body and hemolymph cDNA samples from the bead-challenged pupae as the two "testers" and the cDNA from the samples of the venom treated pupae as the two "drivers". cDNAs from the testers and drivers were first digested into fragments by *Rsa *I (a four-base-cutting restriction enzyme (Clontech), leaving blunt-ended fragments). Each tester cDNA was then divided into two aliquots, each of which was subsequently ligated with one of two different adaptors, adaptor 1 or adaptor 2R. Two different adaptor-ligated tester cDNAs were separately denatured at 98 °C for 90 s and then hybridized at 68 °C for 8 h with an excess of relative driver cDNA in a ratio of tester: driver = 1: 30. These two primary hybridization samples were mixed together without denaturing, and then fresh denatured driver cDNA was added for the second enrichment, hybridizing at 68 °C for 16 h. After that, two rounds of suppression PCR were performed to selectively amplify the differential transcripts. The first PCR was carried out with primer 1 and the program parameter supplied by the manufacturer. The second PCR with nested primers 1 and 2R was performed using diluted primary PCR products (1/10 dilution) as a template. The following two forward subtractive cDNA products were acquired, which could be used to identify early immune-inducible genes, whose transcription levels were specifically down-regulated by the venom from its parasitoid. The resulting secondary PCR products were cloned into pGEM^® ^T-Easy Vector (Promega, Madison, WI, United States) and transformed into High Efficiency JM109 Competent Cells (Promega). The library was plated on LB agar containing 100 μg/ml ampicillin, 0.5 mM IPTG and 80 μg/ml X-gal and incubated at 37 °C overnight.

Colony PCR was performed randomly using the Nested primer 1 and primer 2R supplied by the Kit. Three microliters of the resulting PCR products were identically spotted onto two sheets of positively charged nylon membranes (Roche, Lewes, UK). The membranes were dried and UV cross-linked using a Bio-Rad UV cross-linker (Bio-Rad, Hercules, CA), according to the manufacturer's instructions. Digoxigenin-labeled probes for hybridization were generated using enough non-subtracted cDNAs by the Dig-High Prime Labeling Kit (Roche). The whole process of the dot-blotting was performed using Dig Easy Hyb Granules, Dig-Wash and Block Buffer Set, Anti-Digoxigenin-AP and NBT/BCIP ready-to-use tablets (Roche) following the manufacturers' instructions.

Depending on the result of dolt-blot hybridization, the positively subtractive clones were randomly subjected to sequencing. The deduced amino acid sequences were matched by searching the GenBank database http://www.ncbi.nlm.nih.gov/, using BLASTX algorithm. InterProScan http://www.ebi.ac.uk/InterProScan/ was used for an integrated search in PROSITE, Pfam, and PRINTS databases at EMBL-European Bioinformatics Institute to check whether the functional domains included in unknown genes, which were first analyzed by BLASTX program.

### Sequence alignments and phylogenetic analysis

The deduced amino acid sequences of *P. rapae *cecropin and lysozyme were translated from their cDNA sequences, and other reported cecropin and lysozyme sequences were retrieved from the GenBank database. Multiple sequence alignments were computed using Clustal W2 http://www.ebi.ac.uk/Tools/clustalw2/index.html. The phylogenetic trees were constructed using the neighbor-joining method with a Kimura collection of distances.

### Expression profiling by semi-quantitative RT-PCR

Some candidate genes, especially related to insect innate immunity were selected for comparing their apparent expression profiles between hemocytes and fat body from immune-challenged and immune-challenged-venom-treated hosts. The host pupae injected with PBS were negative controls. After extraction from the hemocytes and fat body, the total RNA samples were treated with TURBO™ DNase (Ambion, Austin, TX) to remove any genomic DNA contaminants. Two and a half micrograms of the DNA-free total RNA was used to synthesize the first strand cDNA by SuperScript^® ^III First-Strand Synthesis System (Invitrogen). The synthesized cDNA was used as a template for RT-PCR. The sequences of pairs of the PCR primers, expected size of PCR product for each candidate gene, and the program parameters of each RT-PCR are in Additional file 4.

### Enzyme assays

Three enzymes related to host immune responses or detoxification, were chosen for analysis. Fifty pupae from each treatment (immune stimulated, immune stimulated-venom-treated treatments and negative control setting like above) were dissected to obtain hemocytes, plasma, fat body, gut and cuticle. Samples were first homogenized in PBS at 0°C (except plasma), and then centrifuged at 10,000 g for 20 min at 4°C. The supernatants were collected as crude enzyme preparations. Protein quantification was determined by the Bradford method [58] using BSA to create a standard curve.

For determination of PO activity a 1 ml mixture containing 2 mM dopamine (Sigma), 50 mM sodium phosphate buffer (pH 6.0) and 10 mg of crude enzyme protein was incubated at 28 °C and the increase in absorbance at 490 nm was continuously monitored. One unit of enzyme activity was defined as an increase of 0.001 in absorbance/minute/mg protein.

Lysozyme activity was measured with a continuous spectrophotometric assay, using lyophilized *Micrococcus luteus *cells (Sigma) as the substrate, as described previously [59]. One unit of enzyme activity was defined as an increase of 0.001 in absorbance at 450 nm/minute/mg protein.

GST activity was measured with the method described by Kao et al [60] with little modification. Briefly, the mixture containing 50 ml (10 mg) crude of enzyme solution, 1.83 ml Tris-HCl buffer (0.1 M, pH 8.0) and 100 μl reduced glutathione (GSH, 50 mM, Sigma). After pre-incubation for 5 min at 25 °C, 20 μl of 1-chloro-2, 4-dinitrobenzene (CDNB) (10 mM, Fluka, Buchs, Italy) was added. The change in optical density was recorded at 340 nm and one unit of enzyme activity was defined as μmol/minute/mg protein.

### Statistic analysis

The data on encapsulation was analyzed for two different treatments (venom-treated and non-treated) and sampling time post treatments by two-way analysis of variance (ANOVA), and all percentages were first transformed to a quasi-normal distribution by arcsine-square root values prior to the statistic analysis. Data on the activity of each enzyme among different treatments (immune-inducible, suppressed, and non-treated) were analyzed by one-way ANOVA, in each tissue tested. Means were compared using LSD tests in all analysis mentioned above. All statistical calculations were performed by DPS software (version 8.01) [61] and statistical significance was set at *P *< 0.05.

## Authors' contributions

QF conducted the major part of this study including experimental design, sampling for RNA isolation, constructions of the subtractive cDNA libraries, EST analysis, RT-PCR analysis, and manuscript preparation. LW participated in insect rearing, preparation of the parasitoid venom, and enzyme assays. JYZ assisted in the development of the project and EST analysis. YML participated in venom preparation and the *in vivo *encapsulation assay. QSS and DWS contributed to provide some valuable suggestions for the experiments and prepare the manuscript. ZRA participated in manuscript preparation. GYY contributed to conceive and design the experiments, coordinate the project, and participate in the manuscript preparation. All authors read and approved the final manuscript.

## Supplementary Material

Additional file 1 **The results of dot blot hybridization**. Dot blot hybridization of colony PCR products from the host hemocytes and fat body forward SSH library, respectively. Click here for file

Additional file 2 **Multiple sequence alignment and phylogenetic analysis for Pr-CecA1-3**. Multiple sequence alignment and phylogenetic analysis between Pr-CecA1-3 identified from SSH library and other cecropins. Click here for file

Additional file 3 **Multiple sequence alignment and phylogenetic analysis for Pr-Lys**. Multiple sequence alignment and phylogenetic analysis between Pr-Lys identified from SSH library and other lysozymes. Click here for file

Additional file 4 **Primers and the parameters of the RT-PCR**. Shows the sequences of forward and reverse primers, expected size of product, and program parameters of the semi-quantitative RT-PCR for each of 40 candidate genes, which were identified from the two forward subtractive libraries of host pupal hemocytes and fat body. Click here for file
