# Supplementary material for: Expression of immune-response genes in lepidopteran host is suppressed by venom from an endoparasitoid, Pteromalus puparum
Source: BMC Genomics. 2010 Sep 2;11:484. doi: 10.1186/1471-2164-11-484 (PMC2996980; doi:10.1186/1471-2164-11-484)

# Additional File 1

Dot blot hybridization of colony PCR products from the host hemocytes and fat body forward SSH libraries, respectively. mRNA samples from immune-inducible and immune-suppressed hemocytes and fat body were severally labeled by DIG as two sets of probes in reverse transcriptional reactions. The orders of the dot-blot hybridization are presented as “a1” to “h12” while the negative control (no PCR products spotting on the membrane) is denoted as “c”. Hybridization was performed using two probes against the PCR products, respectively. (A): PCR products from hemocyte forward SSH library. (I): Blots with probes made from the immune-inducible tissue mRNA; (II): Blots with probes made from the immune-suppressed tissue mRNA. (B): From fat body forward SSH library. The description of (I) and (II) is the same as (A).


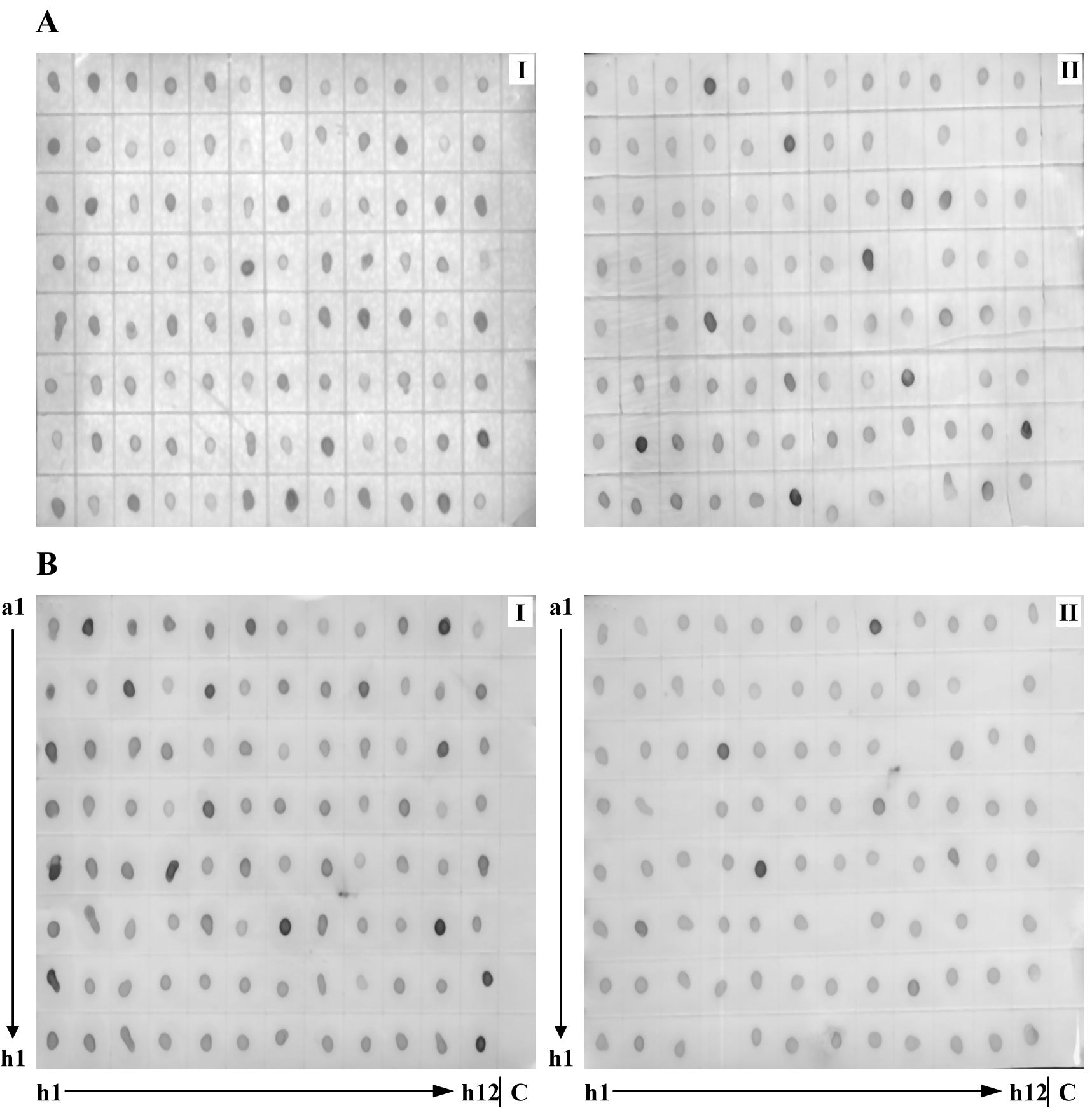

Supplement: Additional file 1 — The results of dot blot hybridization. Dot blot hybridization of colony PCR products from the host hemocytes and fat body forward SSH library, respectively. [file 1471-2164-11-484-S1.DOC]
