# Supplementary material for: Expression of immune-response genes in lepidopteran host is suppressed by venom from an endoparasitoid, Pteromalus puparum
Source: BMC Genomics. 2010 Sep 2;11:484. doi: 10.1186/1471-2164-11-484 (PMC2996980; doi:10.1186/1471-2164-11-484)

# Additional File 2

Multiple sequence alignment and phylogenetic analysis between Pr-CecA1-3 identified from SSH library and other cecropins. (A): Comparison of the amino acid sequences of *P*. *rapae* cecropins (PrcecA1-3) with typical lepidopteran cecropin precursors (containing the signal peptide of each cecropin). The boxes with the same color indicate the same amino acid residues in those comprised species. Spots indicate gaps to optimize the sequence alignment. (B): Phylogenetic analysis of cecropins supplied. Constructions are performed on the basis of the homology sequences calculated from the complete amino acid sequences of cecropin precursors (cec). Sequences are selected from NCBI databases. The amino acid sequences of cecropins of nematode ascaris cecropins (AscecP1-4) are used as the out-group. The box implied the positions of the PrcecA1-3 in the Phylogenetic tree. The sequences used are listed in the table below.

**The sequences of cecropins, used in multiple alignments and phylogenetic tree constructions.**

| **Sequences of cecropin** | | | | | |
| --- | --- | --- | --- | --- | --- |
| **Name** | **Accession No.** | **Species** | **Name** | **Accession No.** | **Species** |
| PrcecA1 | - | *Pieris rapae* | BmcecCBM1 | NP_001037030 | *Bombyx mori* |
| PrcecA2 | - | *Pieris rapae* | BmcecCBM2 | NP_001037031 | *Bombyx mori* |
| PrcecA3 | - | *Pieris rapae* | BmcecCBM22 | NP_001037032 | *Bombyx mori* |
| ArhinII | AAT94287 | *Artogeia rapae* | BmcecD | NP_001036833 | *Bombyx mori* |
| AscecP1 | BAD89085 | *Ascaris suum* | BmcecE | NP_001037392 | *Bombyx mori* |
| AscecP2 | BAD89086 | *Ascaris suum* | DmcecA1 | NP_524588 | *Drosophila melanogaster* |
| AscecP3 | BAD89091 | *Ascaris suum* | DmcecA2 | NP_524589 | *Drosophila melanogaster* |
| AscecP4 | BAD89092 | *Ascaris suum* | DmcecB | BAA28722 | *Drosophila melanogaster* |
| BmcecA | NP_001037462 | *Bombyx mori* | DmcecC | AAB82507 | *Drosophila melanogaster* |
| BmcecB | NP_001037460 | *Bombyx mori* | Hacec | AAX51304 | *Helicoverpa armigera* |
| HcececA | P01507 | *Hyalophora cecropia* | Mscec6 | CAL25128 | *Manduca sexta* |
| HcececB | P01508 | *Hyalophora cecropia* | PicecA | AAR99379 | *Pseudoplusia includens* |
| HcececD | P01510 | *Hyalophora cecropia* | PxcecA | BAF64473 | *Plutella xylostella* |
| HcucecA | P50720 | *Hyphantria cunea* | PxcecE | BAF36816 | *Plutella xylostella* |
| HcucecA1 | P50721 | *Hyphantria cunea* | TncecA | P50724 | *Trichoplusia ni* |
| HcucecA2 | P50722 | *Hyphantria cunea* | TncecB | ABV68872 | *Trichoplusia ni* |
| HcucecA3 | P50723 | *Hyphantria cunea* | TncecD | ABV68873 | *Trichoplusia ni* |
| Mdcec1 | AAL08023 | *Musca domestica* |  |  |  |


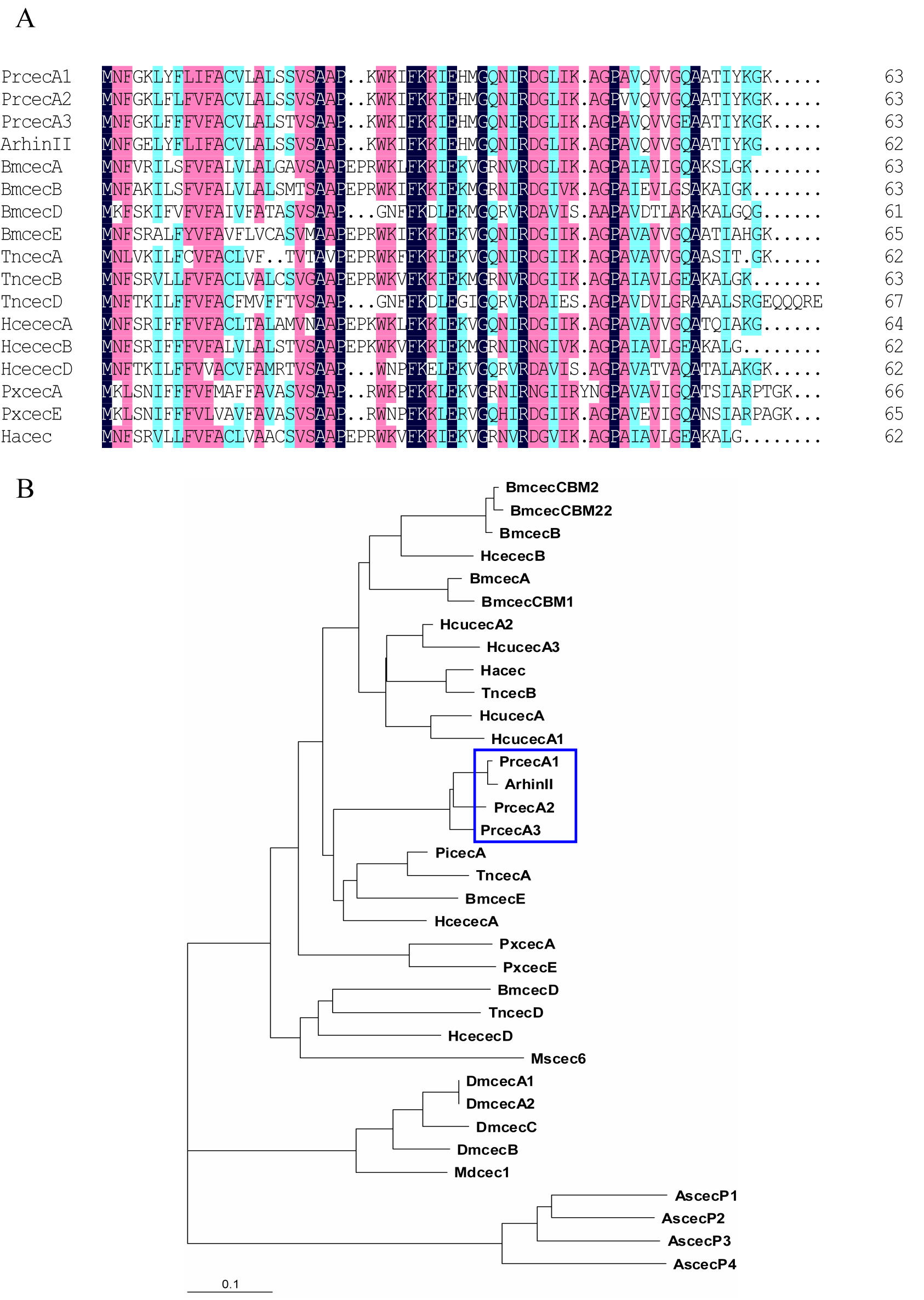

Supplement: Additional file 2 — Multiple sequence alignment and phylogenetic analysis for Pr-CecA1-3. Multiple sequence alignment and phylogenetic analysis between Pr-CecA1-3 identified from SSH library and other cecropins. [file 1471-2164-11-484-S2.DOC]
