# Supplementary material for: Expression of immune-response genes in lepidopteran host is suppressed by venom from an endoparasitoid, Pteromalus puparum
Source: BMC Genomics. 2010 Sep 2;11:484. doi: 10.1186/1471-2164-11-484 (PMC2996980; doi:10.1186/1471-2164-11-484)

# Additional File 3

Multiple sequence alignment and phylogenetic analysis between Pr-Lys identified from SSH library and other lysozymes. (A): Comparison of the amino acid sequences of *P*. *rapae* lysozyme (Pr-Lys) with typical lysozymes (including hen egg). The boxes with same color indicated the same amino acid residues in those comprised species. Spots indicate gaps to optimize the sequence alignment. (B): Phylogenetic analysis of lysozyme supplied. Constructions are performed on the basis of the homology sequences calculated from the complete amino acid sequences of lysozymes (Lys). Sequences are selected from NCBI databases. The amino acid sequence of lysozyme of chicken lysozyme (Ch-Lys) is used as the out-group. The box implies the position of the Pr-LysA in the Phylogenetic tree. The sequences used are listed in the table below.

**The sequences of lysozymes, used in multiple alignments and phylogenetic tree constructions**

| **Sequences of cecropin** | | | | | |
| --- | --- | --- | --- | --- | --- |
| **Name** | **Accession No.** | **Species** | **Name** | **Accession No.** | **Species** |
| Pr-lys | - | *Pieris rapae* | Ha-lys | ABF51015 | *Helicoverpa armigera* |
| Ag-lys | AAC47326 | *Anopheles gambiae* | Hce-lys | P05105 | *Hyalophora cecropia* |
| Ap-lys | ABC73705 | *Antheraea pernyi* | Hv-lys | AAD00078 | *Heliothis virescens* |
| Ar-lysII | AAT94286 | *Artogeia rapae* | Md-lys | ACE00424 | *Musca domestica* |
| Bm-lys | NP_001037448 | *Bombyx mori* | Ms-lys | AAB31190 | *Manduca sexta* |
| Ch-lys | NP_990612 | *Gallus gallus* (Chicken) | Of-lysP | ABN54797 | *Ostrinia furnacalis* |
| Cq-lys | XP_001847112 | *Culex quinquefasciatus* | Pi-lys | AAS48094 | *Pseudoplusia includens* |
| Dm-lysB | NP_523882 | *Drosophila melanogaster* | Scr-lys | BAB20806 | *Samia cynthia ricini* |
| Dm-lysC | NP_524869 | *Drosophila melanogaster* | Se-lys | AAP03061 | *Spodoptera exigua* |
| Dm-lysD | NP_476823 | *Drosophila melanogaster* | Sl-lys | ACI16106 | *Spodoptera litura* |


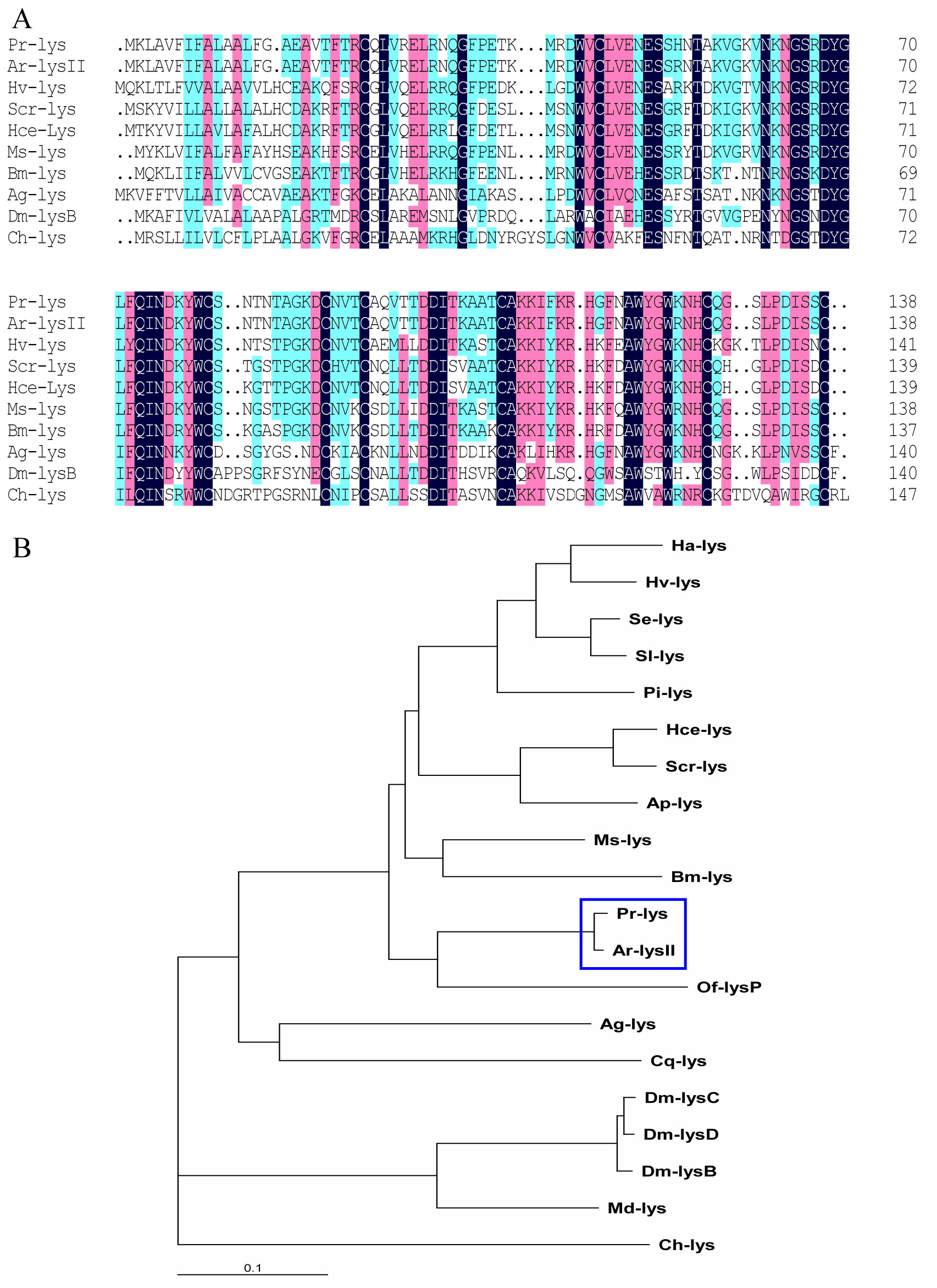

Supplement: Additional file 3 — Multiple sequence alignment and phylogenetic analysis for Pr-Lys. Multiple sequence alignment and phylogenetic analysis between Pr-Lys identified from SSH library and other lysozymes. [file 1471-2164-11-484-S3.DOC]
