# Supplementary material for: Expression of immune-response genes in lepidopteran host is suppressed by venom from an endoparasitoid, Pteromalus puparum
Source: BMC Genomics. 2010 Sep 2;11:484. doi: 10.1186/1471-2164-11-484 (PMC2996980; doi:10.1186/1471-2164-11-484)
Supplement: Additional file 4 — Primers and the parameters of the RT-PCR. Shows the sequences of forward and reverse primers, expected size of product, and program parameters of the semi-quantitative RT-PCR for each of 40 candidate genes, which were identified from the two forward subtractive libraries of host pupal hemocytes and fat body. [file 1471-2164-11-484-S4.DOC]

# Additional File 4

Sequences of PCR primers used to compare the expression profiles by semi-quantitative RT-PCR of the partial genes identified from the two forward subtractive libraries prepared from host pupal hemocytes and fat body.

| **EST ID** | **Primer Sequences (from 5' to 3')** | | **Tm (^o^C)** | **Products** |
| --- | --- | --- | --- | --- |
| **Hemocytes SSH library (Figure 8A)** | | | | |
| hemo-319 | SP | GTTTTCTTATTTACTGTTGGAGG | 53.2 | 305 bp |
|  | AP | TTATCTGGGTAATCTGAGTATCG | 53.9 |  |
| hemo-118 | SP | TTCTCTTTCCCCTTCAGCA | 55.4 | 226 bp |
|  | AP | GGGTTTACGGGCAGTGG | 56.4 |  |
| hemo-15 | SP | GGACAACACTTCCGACAGAGC | 59.4 | 238 bp |
|  | AP | CGTCAACAAAGCCAACATCAT | 58.4 |  |
| hemo-19 | SP | GACACTCTCTTTGAATACTTGGA | 53.4 | 207 bp |
|  | AP | AAATAATGATACTGATTGGGCTA | 53.8 |  |
| hemo-308 | SP | GCAGAACTTCGTCAACTCG | 53.6 | 267 bp |
|  | AP | TACATCAGCCAGAACACTCG | 54.0 |  |
| hemo-11 | SP | ACTTGGTCATCAGTCTTTTGG | 54.1 | 210 bp |
|  | AP | TGTAACACGATTTCTCAACGA | 54.1 |  |
| hemo-232 | SP | AAAGGAAACGCAAGACGAGA | 57.6 | 438 bp |
|  | AP | GTCCGAGCCATAAGCAATCA | 58.5 |  |
| hemo-158 | SP | ACCCTCGCCCAAAGTATCA | 57.7 | 221 bp |
|  | AP | CGGGGAACCTAAGGCAAGT | 59.1 |  |
| hemo-18 | SP | TCTTCGCTTGTGTCTTGGC | 56.7 | 153 bp |
|  | AP | TGTAGATGGTTGCGGCTTG | 57.7 |  |
| hemo-46 | SP | CGCAGTCATCGCATCAATC | 57.7 | 875 bp |
|  | AP | CCTTTTCACAGAAGAACGCAT | 57.2 |  |
| hemo-35 | SP | TTTGCACTTGCTGCTCTGTT | 56.9 | 290 bp |
|  | AP | TTTGTGATGTCGTCCGTTGT | 56.6 |  |
| hemo-176 | SP | AAATCAACGAAAAAACGCA | 54.4 | 139 bp |
|  | AP | CTTGGCAGGTGTCCTTGTT | 55.4 |  |
| hemo-281 | SP | CGTGGTTGGGAAATTAGAA | 53.4 | 135 bp |
|  | AP | GAGAAGCCAGAGTGAGGGT | 53.4 |  |
| hemo-327 | SP | CGCCAGACCCAGACTCACT | 57.8 | 249 bp |
|  | AP | TCCCAGCACAAGCATAACC | 56.5 |  |
| hemo-375 | SP | CGTCAACCAGAGAACCGAG | 55.9 | 271 bp |
|  | AP | CCGAGCATCTACCAGCACA | 57.4 |  |
| hemo-20 | SP | GCGTCTTTTCCAGGGTTAT | 54.3 | 225 bp |
|  | AP | CAGTTGTCGGGTGTTCTCA | 54.1 |  |
| hemo-126 | SP | TAAGATTGACGCAACCGCC | 59.4 | 321 bp |
|  | AP | TCCGCACACATACCATTCG | 58.0 |  |
| hemo-17 | SP | GAGCACGACGAACTGTAAGG | 55.6 | 150 bp |
|  | AP | CGTTCCTGTTTTTGGATTCA | 56.0 |  |
| hemo-371 | SP | TTCAGGCACGAATCTACTCTC | 54.6 | 145 bp |
|  | AP | TTCATTTTTTTGACTTGGCTT | 55.0 |  |
| **Fat body SSH library (Figure 8B)** | | | | |
| FB-212 | SP | GCAGCACGCACTGATTTCCTT | 62.7 | 136 bp |
|  | AP | TGCTCTTTGGTATGAACGGTGAA | 62.3 |  |
| FB-254 | SP | CAGAAACGAGAGAGGACGAT | 54.0 | 312 bp |
|  | AP | GCTACGAAGAAGATGAAATGC | 54.7 |  |
| FB-42 | SP | CACGAGGAGTTCCAACTTCCATAC | 61.9 | 360 bp |
|  | AP | CCCACCACCACTGTCACCATT | 62.4 |  |
| FB-71 | SP | AACCTGCCGATTTTTCTTTG | 57.3 | 253 bp |
|  | AP | TCCTTATCTTGAACCCCACC | 56.4 |  |
| FB-286 | SP | TTGCTTTCACATACGGGACGAT | 62.1 | 166 bp |
|  | AP | TTGTCCACATCAGTGTTCCCTCTT | 62.5 |  |
| FB-183 | SP | GTGTGCCAGTGGACAAAATGA | 58.9 | 180 bp |
|  | AP | AGGTCCACTCCCACTGAAGG | 58.5 |  |
| FB-50 | SP | TGGGTCATCCCTACATTGGCT | 61.6 | 250 bp |
|  | AP | ACCCAGCCCCAAAAATAAACAT | 61.8 |  |
| FB-7 | SP | ACCTTTGTGAAACGCCACTTGA | 62.1 | 277 bp |
|  | AP | TGGGGATACCTTCACACACTCTG | 61.4 |  |
| FB-276 | SP | ACCTACAAAAAGAGCGTCGTGG | 61.2 | 183 bp |
|  | AP | ACGGACTGGCATCTCATCACC | 61.9 |  |
| FB-210 | SP | CACTCCTCCACACAGTTCCCAG | 61.7 | 339 bp |
|  | AP | TTCAACAATCTTCCACTTGCCAT | 61.3 |  |
| FB-70 | SP | AGGAGAGGATTGGATGGTGG | 58.4 | 186 bp |
|  | AP | TTTGCCAGTCTCTTGTCTTCTATG | 58.5 |  |
| FB-20 | SP | AATCGCACCCTGAGGTTGAA | 60.2 | 163 bp |
|  | AP | TCGTAGGCTTTCTTGATTTGAGG | 60.7 |  |
| FB-33 | SP | ATGTCAACACCGAAGCCGAAT | 61.8 | 143 bp |
|  | AP | GAATGGGATGTTTTTGGGTAGGA | 62.1 |  |
| FB-78 | SP | GCGTTTTGATGACTGACTTCTGG | 61.5 | 182 bp |
|  | AP | GTCAACCATTTTCAGAGGCGTG | 62.0 |  |
| FB-101 | SP | GACTCGTGTGTTGGTTGATGGA | 60.4 | 171 bp |
|  | AP | TGGCTAACATCCCATTTTTCG | 60.3 |  |
| FB-95 | SP | CGGAGATAGTGGTATTGGACGG | 61.2 | 108 bp |
|  | AP | GATGTGTGTGAAGAAATCGGGC | 61.8 |  |
| FB-3 | SP | TTCCTTCCATACGGTCAACTGC | 61.6 | 120 bp |
|  | AP | TTCCCAGGCGTCAGTAGGTAGA | 61.4 |  |
| FB-256 | SP | ACTACAGCGGGTATGAGGGAGC | 62.3 | 461 bp |
|  | AP | CACAACTGGCTCAGGTGGTCC | 62.4 |  |
| FB-113 | SP | GCTGGTTCAATGGAAAGATAGAGA | 59.8 | 214 bp |
|  | AP | CACTTGCTGACACATTTTTGGATA | 59.9 |  |
| FB-128 | SP | ATCACGACAAGCACATTTTCTCTG | 61.2 | 234 bp |
|  | AP | ATCAATCTCCTATCGTTCGGCA | 61.3 |  |
| FB-252 | SP | TCTCGCCTGTAGTGCGTGTG | 60.3 | 302 bp |
|  | AP | ACGGTTGTGCTTGTTGGGAG | 60.6 |  |
| 18S rRNA gene | SP | TGAATCTGGATAACTTTTGCC | 54.8 | 170bp |
|  | AP | ATGTGGTAGCCGTTTCTCAG | 55.1 |  |

We used 18 S rRNA gene of *P. rapae* as a standard reference for the semi-quantitative PCR validation, the fragment of which has been screened from another cDNA library built before. The sequence of the fragment of 18 S rRNA gene (430 bp) is listed below.

**18 S rRNA gene sequence:**

**CGGCCAGTGAATTGTAATACGACTCACTATAGGGGGAATTGGGCCCGACGTCGCATGCTCCCGGCCGCCATGGCGGCCGCGGGAATTCGATTTCGAGCGGCCGCCCGGGCAGGTACGCGGGTATTAGATCAAAACCAATCGGCGGAGGGCCTAGCGTCCGAAGTCGTTAATTTTGATGAATCTGGATAACTTTTGCCGATTGCATGGTCCAGTACCGGGGACGCATCTTTCAAATGTTTGCCTTATCAACTTTCGATGATAGTTTTTGCGACTACCATGGTTGTCACGGGTAACGGGGAATCAGGGTTCGATTCCGGAGAGGGAGCCTGAGAAACGGCTACCACATCCAAGGAAGGCAGCAGGCGCGCAAATTACCCACTCCCGGCACGGGGAGGTAGTGACGAAAAAAAAAAAAAAAAAAAAAAAAAAA**

Low Tm value of the pair of primers subtracted by 3 ^o^C are used as the annealing temperature in PCR amplifications, and the Tm values were calculated by the Primer 5.0 software, which were displayed in the table above. For the parameter of the RT-PCR, 29 PCR cycles were used for the amplifications of all genes in Hemocytes SSH library, and 27 PCR cycles for all genes in fat body SSH library, each cycle consisting of 94ºC for 30s, annealing temperature for 60s, and 72ºC for 60s.
